# Mesenchymal stem cell-derived extracellular vesicles prevent the formation of pulmonary arterial hypertension through a microRNA-200b-dependent mechanism

**DOI:** 10.1186/s12931-023-02474-7

**Published:** 2023-09-27

**Authors:** Mengzhi Wan, Caiju Lu, Yu Liu, Feng Luo, Jing Zhou, Fei Xu

**Affiliations:** Department of Respiratory Emergency and Critical Care, The First Affiliated Hospital of Nanchang University, No. 17, Yongwai Zheng Street, Nanchang, Jiangxi Province 330006 P. R. China

**Keywords:** Extracellular vesicles, Bone marrow mesenchymal stem cells, Pulmonary arterial hypertension, microRNA-200b, Macrophage polarization, Luciferase

## Abstract

**Background:**

Bone marrow mesenchymal stem cell-derived extracellular vesicles (BMSC-EVs) have been highly studied with their critical roles as carriers of therapeutic targets such as microRNAs (miRNAs) in the treatment of human diseases, including pulmonary arterial hypertension (PAH). Herein, we tried to study the potential of BMSC-EVs to deliver miR-200b for the regulation of macrophage polarization in PAH.

**Methods:**

Rat models of PAH were induced with monocrotaline treatment, followed by miR-200b expression detection in lung tissues, pulmonary artery smooth muscle cells (PASMCs) and macrophages. miR-200b-containing BMSCs or miR-200b-deficient BMSCs were selected to extract EVs. Then, we assessed the changes in rats with PAH-associated disorders as well as in vitro macrophage polarization and the functions of PASMCs after treatment with BMSC-EVs. Moreover, the interaction between miR-200b, phosphodiesterase 1 A (PDE1A) was identified with a luciferase assay, followed by an exploration of the downstream pathway, cAMP-dependent protein kinase (PKA).

**Results:**

miR-200b was reduced in lung tissues, PASMCs and macrophages of rats with PAH-like pathology. BMSC-EVs transferred miR-200b into macrophages, and subsequently accelerated their switch to the M2 phenotype and reversed the PAH-associated disorders. Furthermore, miR-200b carried by BMSC-EVs induced PKA phosphorylation by targeting PDE1A, thereby expediting macrophage polarization.

**Conclusion:**

Our current study highlighted the inhibitory role of BMSC-EV-miR-200b in PAH formation.

**Supplementary Information:**

The online version contains supplementary material available at 10.1186/s12931-023-02474-7.

## Background

Pulmonary arterial hypertension (PAH) is characterized by a sustained increase in precapillary pulmonary artery pressure [1], resulting from endothelial dysfunction and uncontrolled proliferation of all vascular wall cell types including endothelial, pulmonary artery smooth muscle cells (PASMCs) and fibroblasts, and consequently develops pulmonary vascular remodeling which further leads to right ventricular failure and poor outcomes [2, 3]. Notably, immune cells such as macrophages typically aggregate around pulmonary vessels in PAH [4]. Macrophages confer an important role in pulmonary vascular pathobiology, and even trigger vascular injury by promoting apoptosis of endothelial cells, as well as enhancing smooth muscle cell hypertrophy and proliferation in PAH [4, 5]. Although classified as a rare condition with a prevalence of 20 per 1,000,000 individuals [6], PAH represents a severe clinical condition due to the ineffectiveness of current treatments, and thus warrants additional research to unravel the associated pathophysiological mechanisms [7].

Interestingly, accumulating studies have stressed the paramount importance of extracellular vesicles (EVs) sourced from mesenchymal stem cells (MSCs) as novel therapeutic targets for chronic inflammatory lung diseases, including PAH [8, 9]. Among them, bone marrow-MSCs (BMSCs) possess the ability to alleviate the alterations in pulmonary vascular structure and hemodynamics induced by PAH through paracrine effects [10]. Further adding to their importance, BMSCs modified with insulin-like growth factor binding protein-3 are capable of suppressing the proliferation of PASMCs in PAH [11]. Interestingly, BMSCs can augment nitric oxide production, which underscores the promise for the treatment of PAH which is manifested with reduced endothelial NO release [12]. Accordingly, the current study sought to explore the function and working mechanism of BMSC-EVs during PAH.

EVs can be derived from a wide array of cell types including MSCs, and perform the function of cargo transfer such as DNA, proteins/peptides, mRNA, microRNAs (miRNAs), and lipids to receptor cells [13, 14]. Moreover, one particular miRNA, namely miR-200, can be transferred by EVs from primary human bronchial epithelial cells or MSCs in various pulmonary diseases [15]. Upcoming studies have further indicated that miR-200b-3p promotes the differentiation of monocytes into macrophages and the maturation of macrophages by down-regulating the expression of p38IP, thus influencing the immune response [16]. Meanwhile, a prior study demonstrated that phosphodiesterase-1 (PDE-1) inhibition could eliminate macrophage infiltration and thereby attenuating cold-induced pulmonary hypertension [17]. Moreover, PDE1 silencing results in increased proteasome phosphorylation mediated by cyclic adenosine monophosphate (cAMP)-dependent protein kinase (PKA) in heart failure with preserved ejection fraction [18]. PKA, which is pervasively expressed in mammalian cells, was recently validated as an essential regulator for a range of cellular processes owing to its ability to phosphorylate several protein substrates [19]. On the other hand, enhanced PKA activity has been previously indicated as an alleviator for PAH [20].

Herein the current study, PDE1A was initially predicted to serve as a target of miR-200b through the StarBase database. In lieu of these findings, we surmised that BMSC-EVs could influence the function of macrophages through the PDE1A/PKA axis by delivering miR-200b. Hence, the current study set out to evaluate the specific effects of BMSC-EVs on macrophage polarization during PAH and the mechanisms related to the miR-200b/PDE1A/PKA axis, to ultimately provide a promising therapeutic target against PAH.

## Materials and methods

### Ethics statement

The current study was approved by the Animal Ethics Committee of The First Affiliated Hospital of Nanchang University. All animal experiments were performed in strict accordance with the recommendations for the Guide for the Care and Use of Laboratory Animals of the Ministry of Science and Technology of the People’s Republic of China. Extensive efforts were undertaken to minimize the suffering of the included animals.

### Microarray-based analysis

Firstly, the miRNA expression microarray GSE21284 and gene expression microarray GSE113439 related to PAH were retrieved from the Gene expression omnibus (GEO) database; microarray-related samples and platform annotation files are illustrated in Supplementary Table 1. Differential analyses were conducted using GEO2R [21], and the differentially expressed miRNAs and genes were screened with a threshold of *|log2 fold change (FC)| > 1, p < 0.05*. Functional enrichment analysis of miRNA candidates was carried out with the miRNA similarity (MISIM) database [22]. The downstream targets of miRNA candidates were further predicted using the StarBase database [23], and the genes related to PAH were predicted with the GeneCards database [24]. The predicted target genes, PAH-related genes and up-regulated genes in the microarray GSE113439 using the Jvenn tool [25] were intersected to obtain gene candidates. Afterwards, the Phenolyzer tool [26] was utilized to analyze the interaction network between candidate genes in PAH, and the core interaction genes were selected for further analysis.

### Animal treatment

Male Sprague-Dawley rats (aged 4 weeks, weighing 150–200 g) were procured from Shanghai SLAC Laboratory Animal Co., Ltd. (Shanghai, China). The obtained rats were housed under specified conditions with temperature of 20 − 24 °C and humidity of 45 − 50%, with a 12 h/12 h light/dark cycle and *ad libitum* access to food and water. The rats were subcutaneously administrated with a single dose of monocrotaline (MCT, 40 mg/kg, C2401, Sigma-Aldrich, St. Louis, MO) to induce PAH, or administrated with equal volumes of normal saline as a control and fed normal diet for three weeks. After three weeks, the rats were anesthetized. The PAH model was identified basing on pulmonary artery systolic pressure. Later, the rats were all euthanized by means of CO_2_ inhalation, after which the lungs were collected and quick-frozen for reverse transcription quantitative polymerase chain reaction (RT-qPCR) to analyze the gene expression patterns.

### Isolation, culture and identification of primary PASMCs

Lung tissues of control SD rats and PAH rats were collected to isolate PASMCs, respectively. Next, the internal pulmonary artery at the second branch with a diameter of 100–400 μm was dissected, and the adventitia and endothelium were separated with scissors and cotton swabs in cold phosphate buffered saline (PBS). Subsequently, the artery was cut into small pieces and detached in 0.25% trypsin (25,200,056, GIBCO BRL, Grand Island, NY) at 37 °C for 30 min. Afterwards, the dissociation solution was removed after centrifugation at 200 × g for 5 min at 4 °C and the pellets were resuspended with 5 mL Dulbecco’s modified eagle medium (DMEM, 11,965,902, GIBCO BRL).

The collected cells were cultured in DMEM (GIBCO BRL) containing 10% fetal bovine serum (FBS; 10,091,155, GIBCO BRL) in a 25 cm^2^ culture flask with humidified air of 5% CO_2_ at 37 °C for 3 d -5 d. Next, morphological observation of PASMCs was performed under a phase contrast microscope and the purity was evaluated using immunofluorescence staining with selective anti-α-smooth muscle actin antibody (sc-53,142, Santa Cruz Biotechnology, Santa Cruz, CA). The cells at passage 3 to 5 were used for follow-up experimentation.

### Isolation of alveolar macrophages

Rat lungs were collected after euthanasia, and hearts were perfused with 10 mL of PBS containing 50 U/mL heparin, and then placed in DMEM containing 10% FBS, 10 mM N-2-hydroxyethyl-piperazine-N’-2-ethanesulfonic acid, minimum non-essential amino acids, 2 mM L-glutamine and penicillin/streptomycin solution (1 U/mL penicillin and 1 µg/mL streptomycin in 0.9% NaCl) (cDMEM). Next, the lung lobes were enzymatically hydrolyzed using collagenase XI and IV bovine trypsin at 37 °C, with 5% CO_2_ and 95% relative humidity for 30 min. Subsequently, the obtained cells were suspended into a single cell suspension, and then subjected to mechanical dissociation processes using a gentleMACS dissociator (Miltenyi Biotech GmbH, Bergisch Gladbach, Germany). The enzyme activity was repressed with the addition of cDMEM, and the lung slices were compressed through a sterile 70 μm nylon mesh screen (352,350, BD Biosciences, San Jose, CA) to obtain a single cell suspension. The remaining red blood cells were lysed using 2 µL lysis buffer (0.15 µM NH_4_Cl, 1 µM KHCO_3_) at room temperature for 3 min, and then rinsed with cDMEM. Afterwards, the cells were centrifuged at 4 °C for 7 min at 200 × g, and then resuspended in cDMEM. On average, 8.8 × 10^6^ and 9.9 × 10^6^ lung macrophages were harvested from each control SD rat and PAH rat, respectively. The living cells were counted using the trypan blue exclusion method (97% viability).

### Culture and identification of rat BMSCs

BMSCs utilized for experimentation were isolated from bone marrow aspirates of 4-week-old male rats. A 10 mL syringe was used for aspiration and the needle was carefully rotated after aspiration of every 2 mL bone marrow to minimize peripheral blood contamination. Next, the cells were cultured on a culture plate (where tissues were treated) in Roswell Park Memorial Institute-1640 medium supplemented with 10% FBS and penicillin/streptomycin at 37 °C in humid air containing 5% CO_2_ for 48 h. Upon reaching 80 − 90% cell confluence, BMSCs were trypsinized for subculture. After that, BMSCs at passage no more than 5 were utilized for the remaining experiments. Immunophenotypic characteristics of BMSCs were analyzed by means of flow cytometry. Specifically, the BMSCs were trypsinized for 2 − 4 min, and blocked with 10% normal goat serum (ZSGB-BIO, Beijing, China) to prevent non-specific binding. Afterwards, the BMSCs were cultured with dilutions (1 : 100) of fluorescein isothiocyanate-marked monoclonal antibodies against CD14, CD19, CD29, CD34, CD44, CD45, CD73, CD90, human leucocyte antigen (HLA)-A, HLA-B, HLA-C and human leukocyte antigen-antigen D related (HLA-DR) (BioLegend, San Diego, CA) for 30 min. Later, the BMSCs were resuspended with 10% normal goat serum, and analyzed with a CyAn ADP Analyzer (Beckman Coulter, Miami, FL).

### Identification of rat BMSC differentiation

BMSCs were seeded in a 6-well culture plate, 1 × 10^5^ cells/well. After the cells adhered to the wall, the medium was replaced with osteogenic differentiation medium (DMEM + 0.17 mM vitamin C + 0.5% FBS + 10 mM β Glycerol phosphate + 100 nM dexamethasone + 1% penicillin/streptomycin, Sigma-Aldrich). Next, the cells were allowed to culture for 21 d − 28 d, and the medium was replaced with a new one every 2 d. When the calcified nodules could be observed under the optical microscope, the cells were stained with Alizarin red S (130-22-3, Shanghai Yingxin laboratory equipment Co., Ltd, Shanghai, China) and visualized under an optical microscope.

Following adherence to the wall, the cells were allowed to culture in adipocyte differentiation medium (low-glucose DMEM + glutamine + 10% FBS + 1 µM rosiglitazone + 1 µM dexamethasone + 0.5 mM 3-isobutyl-1-methyl-xanthine + 10 µg/mL insulin + 0.2 mM indomethacin + 1% penicillin/streptomycin). After 3 d, the cell culture continued with low-glucose DMEM supplemented with glutamine, 10% FBS, 1% penicillin/streptomycin, 1 mM rosiglitazone and 10 mg/mL insulin, and the medium was changed every other day. The cells were allowed to culture at 37 °C with 5% CO_2_ for 21 d -28 d. When fat droplets were visible under an optical microscope, the cells were stained with Oil Red O and visualized with an optical microscope.

BMSCs were then seeded in a 15 mL centrifuge tube at a density of 2 × 10^6^, cultured at 37 °C with 5% CO_2_ for 24 h, and then further cultured in a chondrogenic differentiation medium [DMEM (4.5 g/L glucose) + 100 nM dexamethasone + 0.35 mM proline + 0.17 mM vitamin C + 1 mM sodium pyruvate + 1% insulin-transferrin-selenium + 10 ng/mL TGFβ-3 + 1% penicillin/streptomycin (Sigma-Aldrich)] at 37 °C with 5% CO_2_ for 21 d − 28 d, and the medium was replaced with a new one every 2 d. After the cells became a sphere with 1.5 − 2 mm in diameter, the cells were sliced and the slices were stained with Alcian Blue and visualized under an optical microscope.

#### Identification of BMSC-EVs

The size distribution of BMSC-EVs was analyzed by dynamic light scattering (DLS) using a Nanosizer™ instrument (Malvern Panalytical, Worcestershire, UK). Briefly, the BMSC-EVs were diluted in 1 mL PBS and the particle size was automatically tracked and determined depending to the Brownian motion and diffusion coefficient. Next, the morphological observation of BMSC-EVs was done using a Hitachi H-7650 transmission electron microscope (TEM, Hitachi, Tokyo, Japan). Subsequently, 10 µL BMSC-EVs were placed on a 200-mesh copper electron microscope grid coated with Formova carbon, incubated at room temperature for 5 min, and then subjected to staining with standard 1% uranyl acetate at room temperature for 1 min. The expression patterns of BMSC-EV specific surface markers CD63 (ab216130, 1 : 2000, Abcam, Cambridge, UK, rabbit), TSG101 (ab125011, 1 : 10,000, Abcam, rabbit), CD81 (ab109201, 1 : 10,000, Abcam, rabbit) and Calnexin (ab92573, 1 : 100,000, Abcam, rabbit) were determined by means of Western blot analysis to identify the characteristics of BMSC-EVs.

### Uptake of BMSC-EVs

To determine the uptake of BMSC-EVs by PASMCs or alveolar macrophages, BMSC-EVs were marked with a green fluorescent dye (PKH67, Sigma-Aldrich), and incubated together with PAMSCs or alveolar macrophages at 37 °C for 4 h. Next, the mixture was fixed with 4% paraformaldehyde for 15 min, and the nucleus was stained with 6-diamidino-2-phenylindole (DAPI, D1306, 0.5 mg/mL, Invitrogen, Carlsbad, CA) in conditions void of light for 10 min. Subsequently, the green fluorescence of PAMSCs and alveolar macrophages were visualized under a fluorescence microscope. In order to detect the transfer of miRNA from BMSC-EVs to PAMSCs and alveolar macrophages, BMSC-EVs were collected to stimulate the recipient cells for 4 h, and the expression patterns of miR-200b were analyzed by RT-qPCR.

### Cell transfection

PAMSCs and alveolar macrophages were transfected with miR-200b mimic, miR-200b inhibitor, siRNA targeting PDE1A (si-PDE1A), PDE1A over-expression plasmid (pcDNA-PDE1A) and their negative controls (mimic-NC, inhibitor-NC, si-NC, pcDNA-3.1) in compliance with provided specifications for the Lipofectamine 3000 reagent (L3000015, Invitrogen). All above-mentioned plasmids were purchased from Ribobio (Guangzhou, Guangdong, China).

To determine whether BMSC-EVs could deliver miR-200b into macrophages, BMSCs were transfected with Cy3-labeled miR-200b (Cy3-miR-200b), and then co-cultured with macrophages for 48 h. Next, GW4869 was utilized to block the release of EVs from BMSCs prior to co-culture. Afterwards, the PAMSCs and alveolar macrophages were stained by DAPI. Finally, the internalization of BMSC-EV-miR-200b was visualized using a confocal microscope.

Prior to EV isolation, the BMSCs were transfected with miR-200b inhibitor, miR-200b mimic or their NCs (inhibitor-NC, mimic-NC). The isolated EVs were designated as BMSC-miR-200b inhibitor-EVs, BMSC-miR-200b mimic-EVs, BMSC-inhibitor NC-EVs, and BMSC-mimic NC-EVs.

PAMSCs and alveolar macrophages were then co-cultured with BMSC-EVs, BMSC-inhibitor NC-EVs, BMSC-inhibitor NC-EVs, BMSC-EVs + pcDNA-3.1, or BMSC-EVs + pcDNA-PDE1A.

### Cell counting kit 8 (CCK8) assay

Viability of PASMCs after being subjected to different transfection was determined employing a CCK-8 kit (CK04-11, Dojindo, Kyushu Island, Japan). In short, the cells were seeded in a 96-well plate, 3 × 10^3^ cells/well, and 10 µL of CCK8 solution was supplemented to each well at the 0 h, 24 h, 48 h, and 72 h time intervals, respectively, for another 2 h of incubation at 37 °C. Later, the optical density (OD) values at 450 nm were detected using a microplate reader (Molecular Devices, San Jose, CA).

### Flow cytometry

Apoptosis of PASMCs after being subjected to different transfection was analyzed by flow cytometry using a BD Accuri™ C6 flow cytometer (BD Biosciences, San Jose, CA) with excitation at 488 nm. Fluorescence signals from FITC- and propidium iodide (PI)-stained cells were collected at the 530/30 nm and 585/40 nm emission channels, respectively. Cell counting data were analyzed using the FCS Express V4 software (De Novo Software, Glendale, CA).

### RT-qPCR

Total RNA content was extracted from cells and tissues using the Trizol Reagent (15,596,026, Invitrogen). A revert Aid first-strand cDNA synthesis kit (K1621, Thermo Fisher Scientific, Waltham, MA) was utilized to synthesize cDNA by reverse transcription. Next, real-time qPCR was done using SYBR Premix ExTaq^TM^II equipped with an ABI PRISM® 7900HT System (RR420A, Takara, Tokyo, Japan). With GAPDH serving as a reference, the relative standard curve method (2^−ΔΔCT^) was adopted to determine the relative mRNA expression. Primers for this experimentation are illustrated in Supplementary Table 2. BMSC-EV-miRNA was isolated using a SeraMir EVome RNA Purification kit (484,889, System Biosciences, Mountain View, CA). cDNA was synthesized in accordance with the provided instructions with the TaqMan microRNA assay kit (10,524,815, Thermo Fisher Scientific). Using universal reverse primers, real-time qPCR assay was performed using the FastStart Universal SYBR Green Master Mix (Roche, Basel, Switzerland) with the miRNA-specific forward primer (Sangon Biotech, Shanghai, China) and the TaqMan microRNA assay kit. U6 small RNA was adopted as the internal control for miRNA.

### Western blot analysis

Following sodium dodecyl sulfate-polyacrylamide gel electrophoresis, the separated proteins were transferred to a polyvinylidene fluoride membrane (Immobilon P, Merck Millipore, Billerica, MA). Next, the membrane was blocked with tris-buffered saline containing 5% skim milk and 0.1% Tween-20 for 1 h at room temperature. Subsequently, the membrane was incubated with primary antibodies interleukin 6 [IL-6, #12,912, Cell Signaling Technology (CST, Danvers, MA)], tumor necrosis factor alpha (TNF-α, #3707, CST), interleukin-1β (IL-1β, #12,242, CST), PKA (#4782, CST), p-PKA (phospho S99) (ab238951, abcam), myo-inositol-1-phosphate synthase (iNOS, #39,898, CST), arginase 1 (Arg1, #93,668, CST), and PDE1A (PD1A-101AP, Fabgennix International, Frisco, TX, anti-rabbit) overnight at 4 °C. The following day, the membrane was incubated with horseradish peroxidase-labeled secondary antibodies anti-rabbit immunoglobulin G (IgG, #7074) or anti-mouse IgG (#7076) at 37 °C for 1 h. The immune response bands were visualized with an enhanced chemiluminescence reagent (Thermo Fisher Scientific), and photographed using a ChemiDoc XRS Plus luminescent image analyzer (Bio-Rad, Hercules, CA). The band intensity of GAPDH (AC033, Abclonal, Wuhan, Hubei, China, anti-mouse) was adopted as the reference.

### Dual-luciferase reporter gene assay

Human PDE1A 3’-UTR sequence (UGCAUUUAUUGUAUUUUAAGAUAU) containing the predicted miR-200b binding site or PDE1A 3’-UTR mutation sequence (UGCAUUUAUUGUAUUAUUCUACU) was inserted into the pGL3 promoter vector (Genscript, Nanjing, Jiangsu, China). Next, 293T cell (Shanghai Institute of Biochemistry and Cell Biology, Shanghai, China) was seeded in a 24-well plate at the density of 5 × 10^5^ cells/well 1 d prior to transfection. The luciferase reporter vector (0.12 µg) and miR-200b-mimic or miR-200b-NC were co-introduced into 293T cells as per the Lipofectamine 3000 reagent instructions (Invitrogen), and analyzed after 48 h. Luciferase activity was obtained using the luciferase detection kits (RG005, Beyotime, Shanghai, China) on a Glomax 20/20 Luminometer (Promega, Madison, WI).

### Construction of rat model with PAH

Male Sprague-Dawley rats (Shanghai SLAC Laboratory Animal Co., Ltd.) were maintained under climate-controlled conditions with a dark-light cycle of 12 h and *ad libitum* access to food and water for 5 d. The current study adopted the MCT method to establish PAH rat models. Briefly, MCT (Sigma-Aldrich) was dissolved in phosphate buffer, of which the pH value was adjusted to 7.4 using 0.1 N HCl and the volume was increased to reach a final concentration of 20 mg/mL. Next, the MCT solution was sterilized with a 0.45-µm syringe filter, and aliquoted and stored at 20 °C. Rats were subcutaneously injected with MCT (Sigma-Aldrich, 40 mg/kg) or saline (control). The dose of MCT used in this experiment was determined by our pre-experimental work to reduce the high mortality of rats after MCT treatment. Throughout the experiment, food intake per cage was monitored, and 4–5 rats were subjected to similar treatment in each cage.

Rats were randomized into mock, BMSC-EV, BMSC-EV + miR-200b inhibitor, BMSC-EV + inhibitor-NC, BMSCs-EV + pc-DNA-Vector, and BMSCs-EV + pc-DNA-PDE1A groups. Rats in each group received a single subcutaneous injection of MCT (Sigma-Aldrich, St-Louis, MO) (40 mg/kg) or saline control. Twenty-one days after MCT treatment, rats were intraperitoneally injected with 0.2 mL of BMSC-EV-treated cells (3 × 10^6^) every other day. miR-200b inhibitor and its controls, purified plasmids pc-DNA-Vector and pc-DNA-PDE1A were injected intramuscularly. At the 35th d, all rats were examined and PAH-related indicators were measured by means of echocardiography. A 1.2 French pressure catheter (Scisense Inc., Ontario, Canada) was linked to the Scisense FA-404 recorder. After exposing the right jugular vein, the catheter was inserted into the vein, then advanced to the superior vena cava, and finally into the right ventricular vein. The mPAP was recorded and right ventricular systolic pressure (RVSP) values were recorded continuously for 45 min. Following mPAP and RVSP measurement, the chest cavity was opened, and the heart was dissected and weighed to calculate the right ventricular hypertrophy index (RVHI) (the ratio of the weight of the right ventricular free wall to the sum of the diaphragm plus the weight of the left ventricular free wall) using the following formula: RVHI = (RV / LV + S).

### Immunofluorescence staining

Rat lung tissues after being subjected to different treatments were fixed in 10% neutralized formalin and embedded in paraffin blocks. Next, the obtained Sect. (4 μm) were deparaffinized with xylene and rehydrated with gradient ethanol. The cells were then incubated with Triton X-100 (Sigma-Aldrich) to increase their permeability. Following blockade with normal non-immune goat serum for 30 min, the tissue sections were probed with CD68 (MA5-13324, Thermo Fisher Scientific), CD16 (#80,366, CST), CD206 (PA5-101657, Thermo Fisher Scientific) at 37 °C for 2 h. Later, the cells were stained with secondary immunoreagents coupled with Alexa dye, phalloidin coupled with Alexa dye and Hoechst 33,342 (Invitrogen). Afterwards, NC staining was performed by omitting the primary antibody.

### Hematoxylin-eosin (H&E) staining

The left lung lobe was cut and processed longitudinally. The paraffin-embedded tissue samples were sliced into sections with a thickness of 5 μm. Subsequently, the obtained sections were stained with H&E (G1120, Solarbio, Beijing, China) to observe the severity of blood vessels in PAH with a diameter ≤ 50 or 50 − 100 μm, that is, the percentage of hyperplasia in medial pulmonary small vessels.

### Statistical analysis

Statistical analyses were performed using the Statistic Package for Social Science (SPSS) 21.0 statistical software (IBM Corp, Armonk, NY). Measurement data were expressed as *mean ± standard deviation*. Two groups were compared by unpaired *t* test, and multiple groups were compared by one-way analysis of variance (ANOVA), followed by Tukey’s post-hoc test. Time-based comparisons among groups were analyzed by measurement ANOVA, followed by Bonferroni’s post-hoc test. A *p* < 0.05 value was regarded statistically significant.

## Results

### miR-200b was poorly-expressed in lung tissues of rats with PAH

**Fig. 1:**
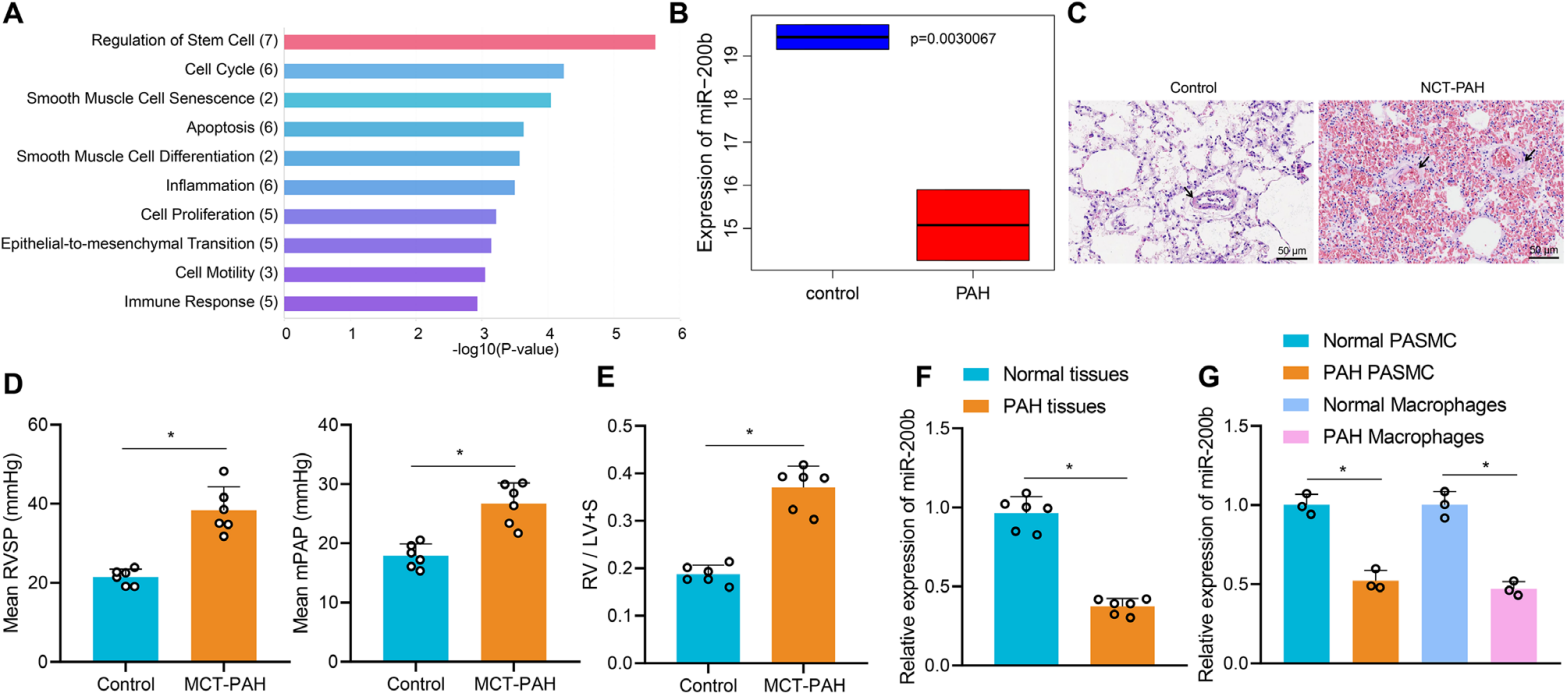
Low expression of miR-200b is detected in the lung tissues of patients with PAH and rats with PAH. **A**, The functional enrichment analysis of miRNAs (the X-axis represents the number of miRNAs involved in the pathway, and the Y-axis represents the pathway name). **B**, The low expression of miR-200b in the patients with PAH in the microarray GSE21284. **C**, Pulmonary artery wall changes in rat models of PAH shown by H&E staining (Scale bar = 50 μm). **D**, The level of RVSP in the rat model of PAH. **E**, The quality of RV in the rat model of PAH determined by RV/(LV + S). **F**, The expression of miR-200b in the lung tissues of rats with PAH and control rats determined by RT-qPCR. **G**, The expression of miR-200b in PASMCs and alveolar macrophages of rats with PAH and control rats determined by RT-qPCR. Comparison between two groups was analyzed by independent sample *t* test, and all experiments were repeated three times

Firstly, a total of 45 differentially expressed miRNAs were obtained from the PAH microarray GSE21284, and subsequent functional enrichment analysis of miRNAs through the MISIM database revealed the involvement of miR-150, miR-143, miR-148a, miR-200b, and miR-223 in the PAH immune response process (Fig. 1A). In addition, the expression of miR-200b in PAH patients was shown to be lower than that of normal controls in the microarray GSE21284 (Fig. 1B). Additionally, rat models of PAH were established by means of MCT treatment. Relative to control rats, the rats treated with MCT presented with thicker pulmonary artery walls, higher mPAP and RVSP, and an increase in the ratio of RV/(LV + S) (Fig. 1C-E), indicating that PAH was successfully induced. Compared with those of control rats, miR-200b levels in the lung tissues of PAH rats were notably down-regulated, while those in the isolated PASMC and alveolar macrophages were also decreased (Fig. 1F, G). Together, these findings indicated that miR-200b was under-expressed in the lung tissues of PAH rats.

### miR-200b was delivered by BMSC-EVs into alveolar macrophages

**Fig. 2:**
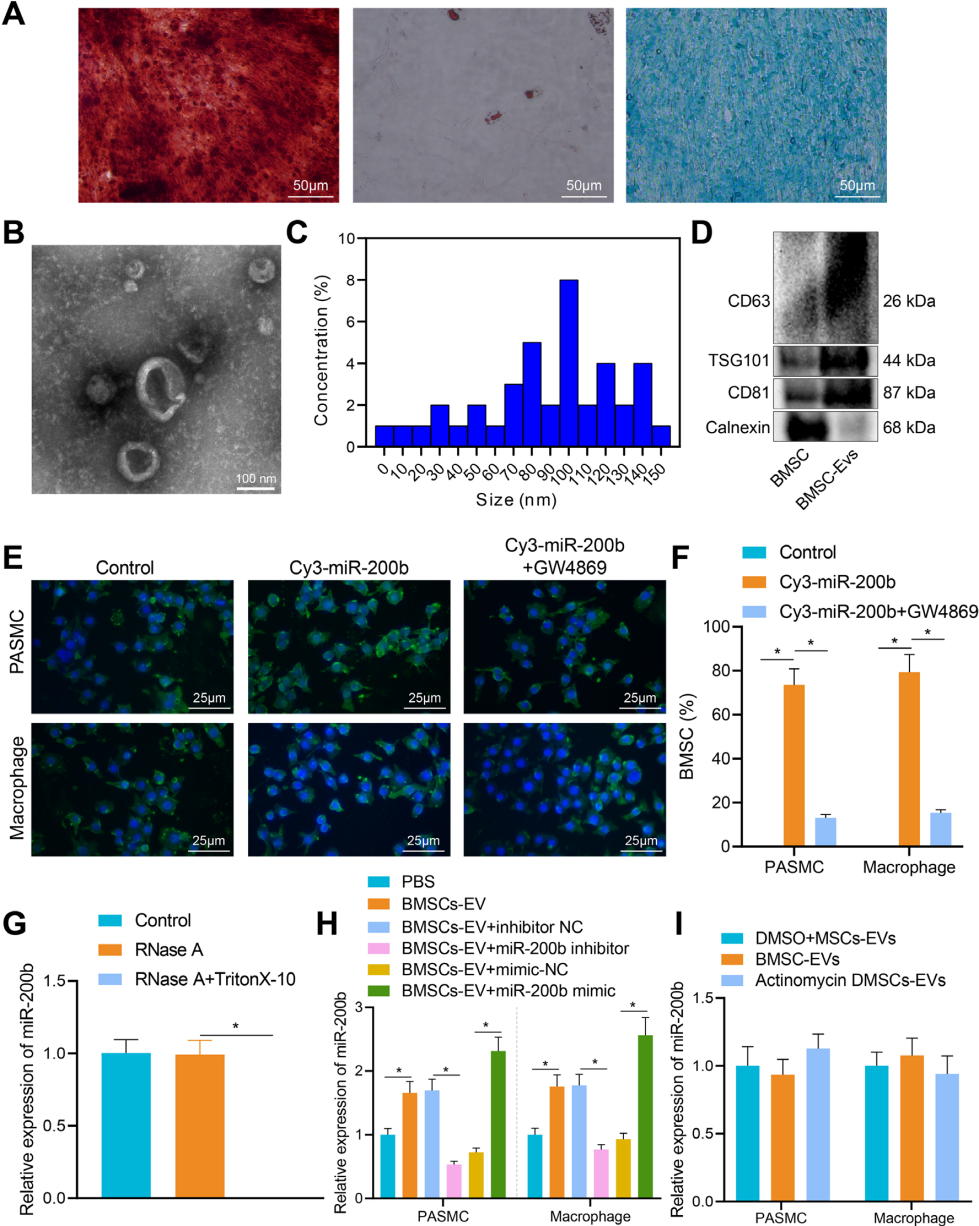
BMSC-EVs transfer miR-200b to alveolar macrophages. **A**, Osteogenic differentiation of BMSCs assessed by Alizarin red staining, adipogenic differentiation of BMSCs assessed by Oil red O staining, and chondrogenic differentiation of BMSCs assessed by Alcian Blue staining (Scale bar = 50 μm). **B**, BMSC-EV morphology observed under a TEM (Scale bar = 100 nm). **C**, The size distribution of BMSC-EVs detected by DLS measurement. **D**, BMSC-EV surface-labeled proteins measured by Western blot analysis. **E**, The internalization of PKH67-labeled BMSC-EVs into the cytoplasm of alveolar macrophages or PASMCs observed by a fluorescence microscope (Scale bar = 25 μm). **F**, The transfer of Cy3-miR-200b into the alveolar macrophages or PASMCs observed by a fluorescence microscope. **G**, The expression of miR-200b in BMSC-EVs determined by RT-qPCR. **H**, The expression of miR-200b in alveolar macrophages or PASMCs after co-culture with different EVs determined by RT-qPCR. **I**, The expression of miR-200b in alveolar macrophages or PASMCs after actinomycin D treatment in the presence of BMSC-EVs determined by RT-qPCR. * *p* < 0.05. Comparison between two groups was analyzed by independent-sample *t*-test, and comparison among multiple groups was analyzed by one-way ANOVA, followed by Tukey’s post hoc test. All experiments were repeated three times

Cell surface markers of isolated cells were detected by means of flow cytometry, the results of which illustrated that the isolated cells were positive for mesenchymal stem cell markers CD90, CD73, and CD105, while being negative for hematopoietic markers CD34, CD19, CD45, CD14, and HLA-DR. Calcium nodule formation was apparent in the cells after osteogenic induction, in addition to lipid droplet accumulation after adipogenic induction of BMSCs. Meanwhile, fibrous-like cells were round- or elliptical-shaped after chondrogenic induction of BMSCs (Fig. 2A). Overall, these observations revealed that the isolated cells exhibited osteogenic, adipogenic and chondrogenic differentiation abilities as BMSCs, indicating the successful isolation of BMSCs.

Next, EVs were separated from BMSC supernatant by means of ultra-centrifugation. Extracted BMSC-EVs exhibited a typical characteristic structure under TEM (Fig. 2B). The size distribution of these nanoparticles was between 20 and 150 nm according to the results of DLS testing (Fig. 2C). In addition, BMSC-EVs were positive for TSG101, CD63, and CD81, while being negative for Calnexin based on the results of Western blot analysis (Fig. 2D). In short, these findings indicated that BMSC-EVs were successfully extracted.

To further examine whether BMSC-EVs could be internalized by alveolar macrophages, PKH67-labeled BMSC-EVs were incubated with PASMCs or alveolar macrophages in vitro. After 24 h of incubation, PKH67-EVs were visualized to be internalized by PASMCs or alveolar macrophages under a fluorescence microscope (Fig. 2E). Next, BMSCs or PASMCs were transfected with Cy3-labeled miR-200b and then co-cultured with macrophages or PASMCs to determine whether BMSC-EVs could deliver miR-200b into macrophages or PASMCs. Subsequent observation revealed that the red fluorescence was dramatically weakened when the BMSCs were treated by GW4869 in the co-culture system (Fig. 2F), indicating that BMSC-EVs could deliver miR-200b into macrophages or PASMCs. In order to verify whether miR-200b was encapsulated in EVs or present on the surface of EVs, an RNase protection experiment was conducted on BMSC-EVs. Following treatment of the conditioned medium of BMSCs with RNase A, RNA content was extracted. It was found that the miR-200b levels in BMSCs remained unchanged after treatment with RNase A alone, while miR-200b levels could not be detected in the BMSCs treated with both RNase A and TritonX-10 (Fig. 2G), indicating that miR-200b was encapsulated by a membrane rather than released directly. Furthermore, BMSCs were treated with miR-200b mimic or miR-200b inhibitor, and then BMSC-EVs (BMSC-miR-200b mimic-EVs and BMSC-miR-200b inhibitor-EVs) were extracted and co-cultured with macrophages. Subsequent RT-qPCR results displayed that miR-200b levels were notably increased in macrophages after co-culture with BMSC-miR-200b mimic-EVs, while being dramatically inhibited in macrophages after co-culture with BMSC-miR-200b inhibitor-EVs; after co-culture with BMSC-EV and PASMC, the same expression trends were also detected (Fig. 2H). To exclude the endogenous induction of miR-200b, macrophages were treated with actinomycin D. RT-qPCR results further illustrated no significant differences in the expression of miR-200b after treatment with actinomycin D in the presence of BMSC-EVs (Fig. 2I), indicating that the expression of miR-200b was not induced endogenously.

Altogether, these findings validated that BMSC-EVs could be internalized by alveolar macrophages or PASMCs, and transmitted miR-200b to alveolar macrophages.

#### BMSC-EVs carrying miR-200b promoted M2 polarization of alveolar macrophages

**Fig. 3:**
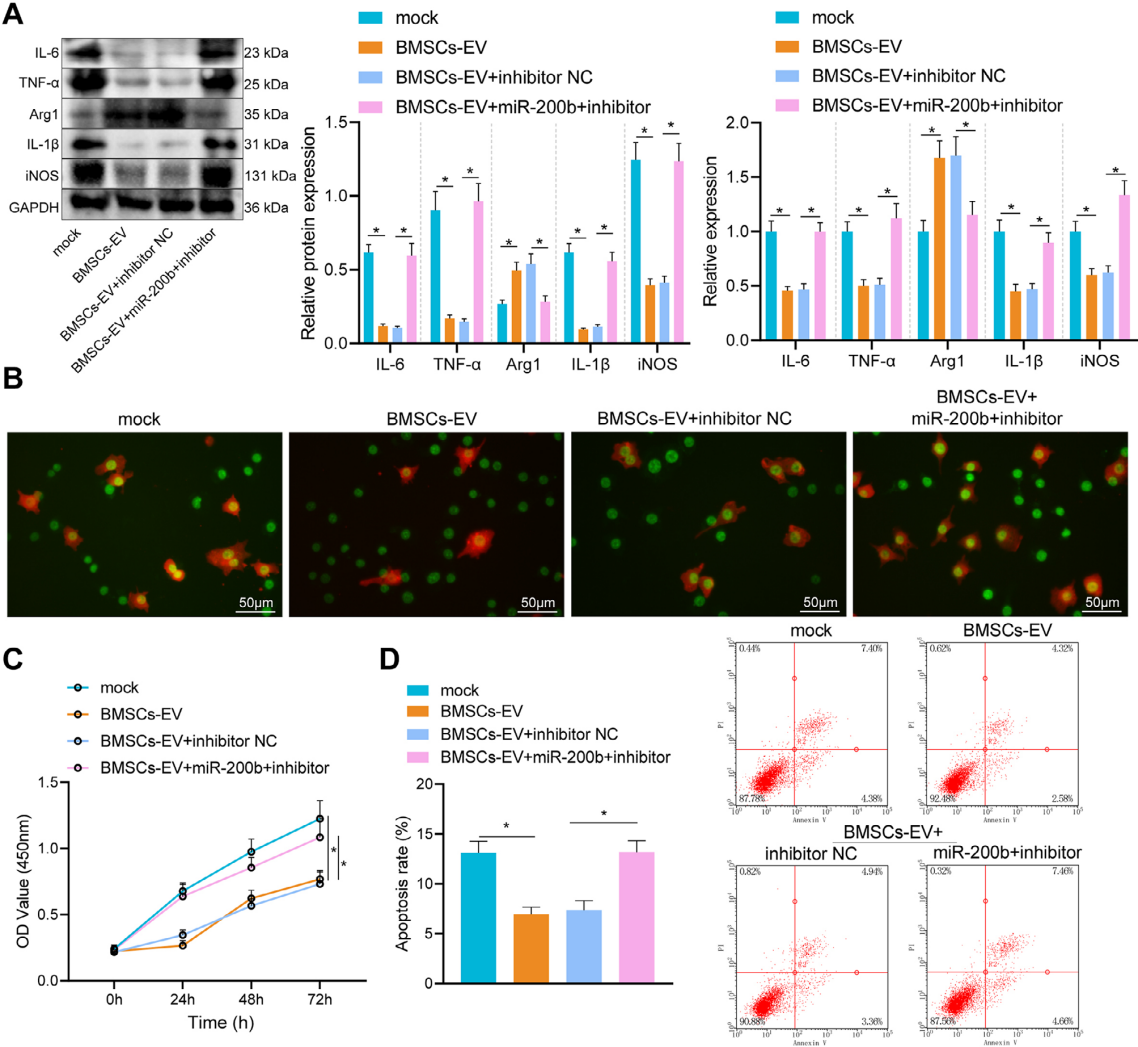
BMSC-EVs carrying miR-200b control the phenotype of macrophages. A, The expression of IL-6, TNF-α, IL-1β, iNOS and Arg1 determined by RT-qPCR and Western blot analysis. B, The polarization of macrophages shown by immunofluorescence staining (Scale bar = 50 μm), the green signal represents Arg1 and the red signal represents iNOS. C, The proliferation of PASMCs evaluated by CCK-8. D, The apoptosis of PASMCs evaluated by flow cytometry. * *p* < 0.05. Comparison among multiple groups was analyzed by one-way ANOVA and followed by Tukey’s post hoc test. All experiments were repeated three times

Thereafter, EVs were extracted from BMSCs transfected with miR-200b mimic or miR-200b inhibitor and then co-cultured with macrophages. Subsequent results demonstrated that the levels of inflammatory factors (IL-6, TNF-α and IL-1β) and M1 macrophage marker (iNOS) were all markedly reduced, while the expression of M2 macrophage marker (Arg1) was sharply increased in macrophages after co-culture with BMSC-EVs, whereas all these changes were reversed upon co-culturing with BMSC-miR-200b inhibitor-EVs (Fig. 3A). Meanwhile, results of immunofluorescence staining illustrated that the proportion of M2 macrophages was notably increased by BMSC-EVs, while the proportion of M1 macrophages was also significantly elevated by BMSC-miR-200b inhibitor-EVs (Fig. 3B). Additional CCK-8 and flow cytometric results uncovered that viability of PASMCs was suppressed and apoptosis was promoted by macrophages co-cultured with BMSC-EVs; whereas, delivery of miR-200b inhibitor by BMSC-EVs into macrophages led to enhanced viability and impeded apoptosis of PASMCs (Fig. 3C, D). To sum up, these findings indicated that BMSC-EVs carrying miR-200b enhanced the M2 macrophage polarization, thereby inhibiting PASMC growth and promoting their apoptosis.

#### BMSC-EVs carrying miR-200b alleviated PAH

**Fig. 4:**
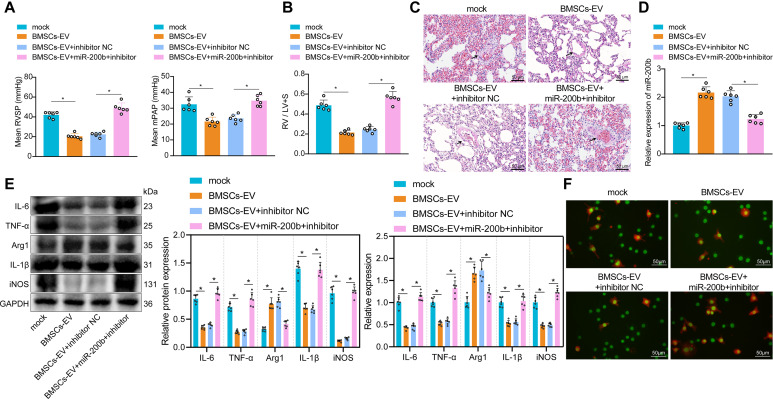
BMSC-EVs carrying miR-200b ameliorate PAH in rats. **A**, RVSP in PAH rats injected with the cells treated with BMSC-EVs alone or combined with miR-200b inhibitor. **B**, RVHI in PAH rats injected with the cells treated with BMSC-EVs alone or combined with miR-200b inhibitor. **C**, Medial thickness of small pulmonary vessels in rats injected with the cells treated with BMSC-EVs alone or combined with miR-200b inhibitor (Scale bar = 50 μm). **D**, The expression of miR-200b in the lung tissues of PAH rats injected with the cells treated with BMSC-EVs alone or combined with miR-200b inhibitor determined by RT-qPCR. E, The expression of IL-6, TNF-α, IL-1β, iNOS, and Arg1 determined by RT-qPCR and Western blot analysis. F, The polarization of macrophages detected by immunofluorescence staining (Scale bar = 50 μm). * *p* < 0.05. Comparison among multiple groups was analyzed by one-way ANOVA and followed by Tukey’s post hoc test. Comparison among groups at different time points was performed by repeated measurement ANOVA and followed by Bonferroni’s post hoc test. All experiments were repeated three times. n = 6

Animal experimentation was further carried out to investigate the impact of miR-200b-containing BMSC-EVs on PAH. As illustrated in Fig. 4A-C, the mPAP, RVSP, RVHI and medial thickness of small pulmonary vessels were all notably reduced in the rats with PAH after injection of BMSC-EVs. Compared with that of PAH rats injected with BMSC-inhibitor-NC-EVs, there was a significant increase in the mPAP, RVSP, RVHI and the medial thickness of the small pulmonary vessels in PAH rats injected with BMSC-miR-200b inhibitor-EVs, indicating that loss of miR-200b reversed the inhibitory effects of BMSC-EVs on PAH. Subsequent results of RT-qPCR demonstrated that miR-200b levels were dramatically increased in the PAH rats injected with BMSC-EVs, while being reduced when PAH rats were injected with BMSC-miR-200b inhibitor-EVs (Fig. 4D). Moreover, the inflammatory factors (IL-6, TNF-α and IL-1β) and M1 macrophage markers (iNOS) in PAH rats were all decreased, while the M2 macrophage marker (Arg1) was increased following treatment with BMSC-EVs, whereas all these changes in mRNAs and proteins were reversed after the loss of miR-200b (Fig. 4E). In addition, immunofluorescence staining illustrated that the proportion of M2 macrophages was significantly increased in the lung tissues of PAH rats injected with BMSC-EVs, while the proportion of M1 macrophages was elevated notably in the lung tissues of PAH rats injected with BMSC-miR-200b inhibitor-EVs (Fig. 4F). Overall, these findings indicated that BMSC-EVs delivered miR-200b to promote M2 macrophage polarization, thereby inhibiting the formation of PAH.

#### miR-200b could target PDE1A

**Fig. 5:**
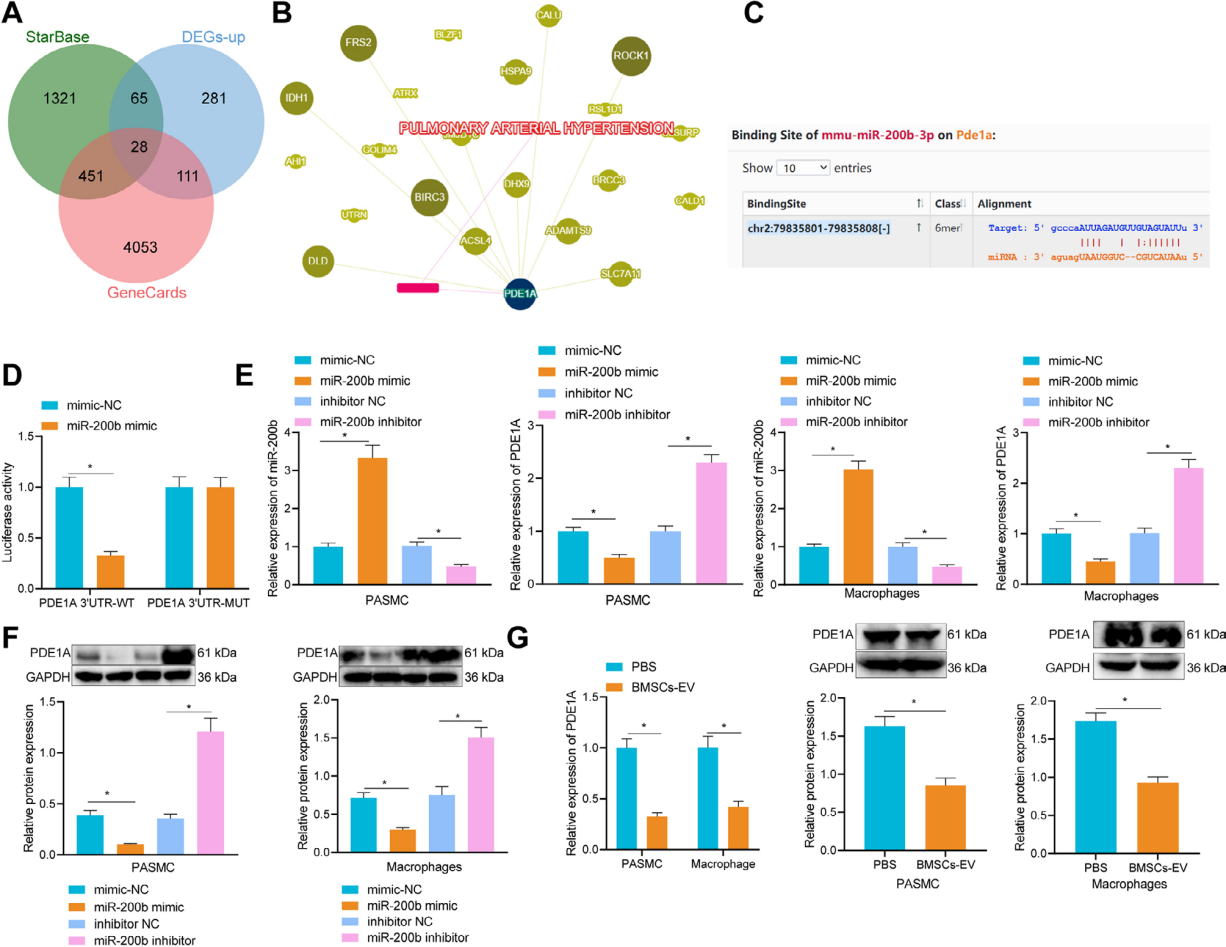
PDE1A expression was negatively regulated by miR-200b. **A**, Venn diagram showing the intersection among upregulated genes in microarray GSE113439, predicted downstream genes of miR-200b, and PAH-related genes. **B**, Interaction network diagram of gene candidates in PAH. **C**, The binding sites of PDE1A and miR-200b predicted by TargetScan. **D**, Luciferase activity of cells in response to miR-200b mimic determined by dual-luciferase reporter gene assay. **E**, The expression of miR-200b and PDE1A in cells in response to miR-200b mimic or inhibitor determined by RT-qPCR. **F**, The expression of PDE1A in cells in response to miR-200b mimic or inhibitor determined by Western blot analysis. **G**, The expression of PDE1A in response to treatment with BMSC-EVs determined by RT-qPCR and Western blot analysis. * *p* < 0.05. Comparison between two groups was analyzed by independent sample-*t* test, and comparison among multiple groups was analyzed by one-way ANOVA followed by Tukey’s post hoc test. All experiments were repeated three times

In an attempt to further elucidate the mechanism underlying BMSC-EVs carrying miR-200b repress the formation of PAH, 1865 downstream targets of miR-200b were retrieved from the StarBase database, and 4643 PAH-related genes were retrieved from the GeneCards database. Thereafter, 28 target gene candidates were obtained at the intersection among predicted downstream targets of miR-200b, PAH-related genes and the up-regulated genes in the microarray GSE113439 (Fig. 5A). An interaction network diagram of the 28 candidate genes in the PAH was subsequently constructed using the Phenolyzer tool, wherein PDE1A was found at the core position, exhibiting a closer relationship with other genes (Fig. 5B). The specific binding sites of miR-200b and PDE1A were further predicted by means of online prediction StarBase (Fig. 5C). Dual-luciferase report gene assay further evidenced that the luciferase activity of PDE1A 3’UTR-WT was reduced by miR-200b mimic, while that of PDE1A 3’UTR-MUT was unchanged (Fig. 5D). The mRNA and protein levels of PDE1A were notably down-regulated after miR-200b gain-of-function in PASMCs and alveolar macrophages by miR-200b mimic, while being up-regulated following miR-200b inhibition (Fig. 5E, F). Meanwhile, compared with those in alveolar macrophages treated with PBS, the mRNA and protein levels of PDE1A in alveolar macrophages treated with BMSC-EVs were markedly reduced (Fig. 5G). Together, these findings highlighted that miR-200b inhibited PDE1A by binding to its 3’UTR.

### miR-200b induced phosphorylation of PKA by inhibiting PDE1A

**Fig. 6:**
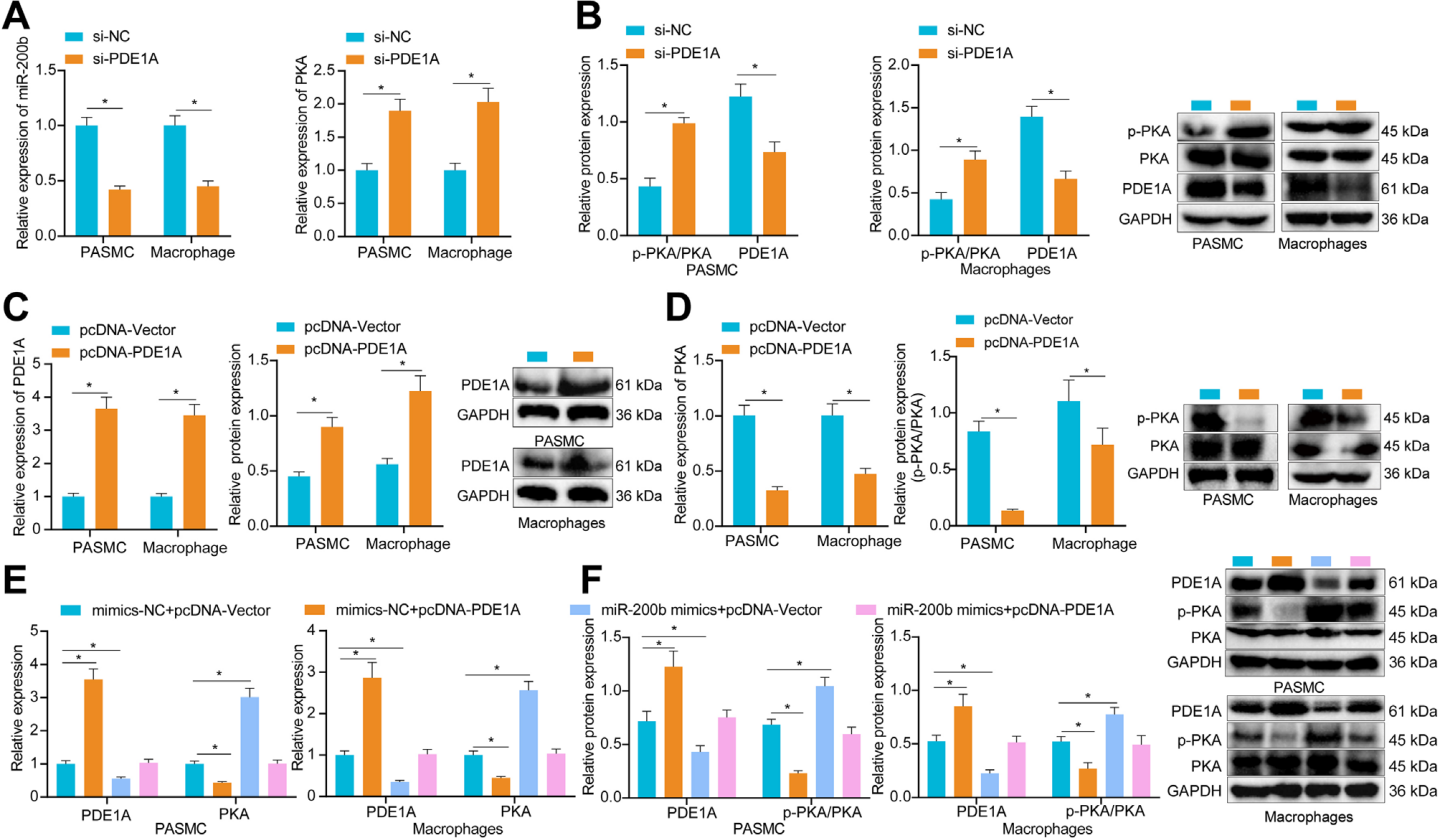
miR-200b targets PDE1A to induce PKA phosphorylation. **A**, The mRNA levels of PDE1A and PKA after treatment with si-PDE1A determined by RT-qPCR. **B**, The protein levels of PDE1A and PKA after treatment with si-PDE1A or pcDNA-PDE1A measured by Western blot analysis. **C**, The mRNA and protein levels of PDE1A after treatment with pcDNA-PDE1A determined by RT-qPCR and Western blot analysis. **D**, The mRNA and protein levels of PKA after treatment with pcDNA-PDE1A determined by RT-qPCR and Western blot analysis. **E**, The mRNA levels of PDE1A and PKA after treatment with miR-200b mimic and/or pcDNA-PDE1A determined by RT-qPCR. **F**, The protein levels of PDE1A and PKA after treatment with miR-200b mimic and/or pcDNA-PDE1A measured by Western blot analysis. * *p* < 0.05. Comparison between two groups was analyzed by independent sample-*t* test, and comparison among multiple groups was analyzed by one-way ANOVA, followed by Tukey’s post hoc test. All experiments were repeated three times

As PDE1 possesses the ability to regulate PKA-mediated proteasome phosphorylation [15], we speculated whether PDE1A functioned in PAH by controlling PKA. It was found that PDE1A levels were down-regulated, while PKA mRNA level and p-PKA/T-PKA level were up-regulated in macrophages treated with si-PDE1A (Fig. 6A, B). On the other hand, the mRNA and protein levels of PDE1A were increased after treatment with pcDNA-PDE1A, while PKA mRNA level and p-PKA/T-PKA level were reduced (Fig. 6C, D), underscoring that PDE1A inhibited the expression of PKA. Furthermore, treatment with pcDNA-PDE1A eliminated the increase in PKA mRNA level and p-PKA/T-PKA level induced by the over-expression of miR-200b (Fig. 6E, F). Overall, these findings established that miR-200b induced the phosphorylation of PKA by repressing PDE1A.

### BMSC-EVs carrying miR-200b attenuated PAH by promoting M2 macrophage polarization through PDE1A/PKA axis

**Fig. 7:**
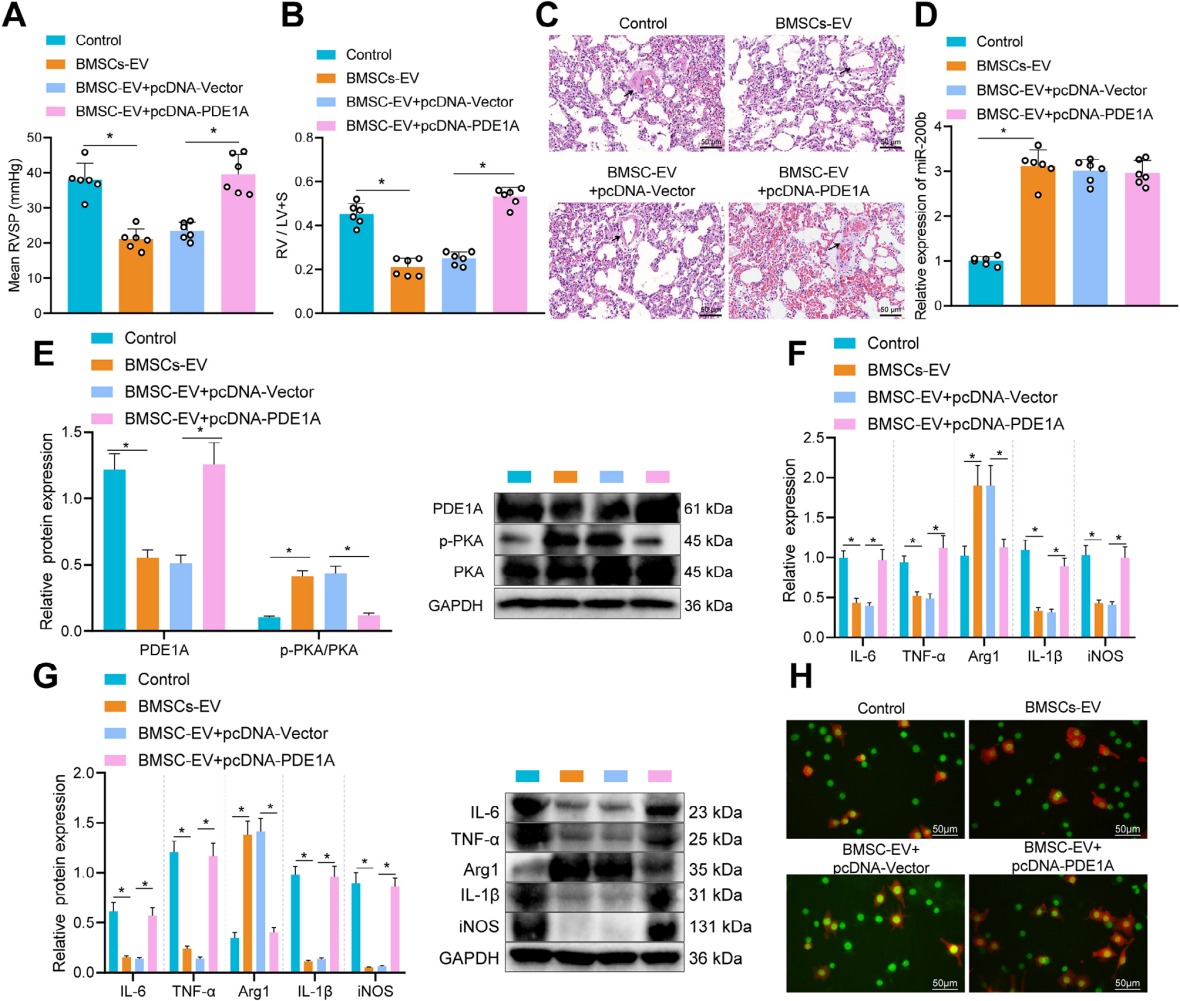
BMSC-EVs carrying miR-200b regulate PDE1A/PKA axis to alleviate PAH in rats. **A**, RVSP in PAH rats injected with the cells treated with BMSC-EVs alone or combined with pcDNA-PDE1A. **B**, RVHI in PAH rats injected with the cells treated with BMSC-EVs alone or combined with pcDNA-PDE1A. **C**, Medial thickness of small pulmonary vessels in PAH rats injected with the cells treated with BMSC-EVs alone or combined with pcDNA-PDE1A (Scale bar = 50 μm). **D**, The expression of miR-200b in the lung tissues of PAH rats injected with the cells treated with BMSC-EVs alone or combined with pcDNA-PDE1A determined by RT-qPCR. **E**, The protein levels of PDE1A and PKA in the lung tissues of PAH rats injected with the cells treated with BMSC-EVs alone or combined with pcDNA-PDE1A measured by Western blot analysis. **F**, The expression of IL-6, TNF-α, IL-1β, INOS and Arg1 determined by RT-qPCR. **G**, The expression of IL-6, TNF-α, IL-1β, INOS and Arg1 measured by Western blot analysis. **H**, The polarization of macrophages shown by immunofluorescence staining (Scale bar = 50 μm). * *p* < 0.05 compared with the PAH rats. Comparison among multiple groups was analyzed by one-way ANOVA and followed by Tukey’s post hoc test. Comparison among groups at different time points was performed by repeated measurement ANOVA, followed by Bonferroni’s post hoc test. n = 6

Lastly, we explored the involvement of miR-200b/PDE1A/PKA axis in the progression of PAH in vivo. Compared with that of PAH rats, the RVSP, RVHI and medial thickness of small pulmonary vessels were all markedly reduced in PAH rats injected with BMSC-EVs. However, these reductions were reversed following treatment with pcDNA-PDE1A (Fig. 7A-C). Meanwhile, miR-200b expression (Fig. 7D) and p-PKA/T-PKA level (Fig. 7E) were both notably elevated, while the protein levels of PDE1A were dramatically reduced in PAH rats injected with BMSC-EVs. Besides, treatment with pcDNA-PDE1A elevated the protein levels of PDE1A, but reduced p-PKA/T-PKA level, indicating that BMSC-EVs carried miR-200b to elevate PKA by targeting PDE1A. In addition, treatment with BMSC-EVs significantly reduced the levels of inflammation markers (IL-6, TNF-α, and IL-1β) and M1 macrophage markers (iNOS) and enhanced the expression of M2 macrophage marker (Arg1); whereas, restoration of PDE1A counteracted these changes induced by BMSC-EVs (Fig. 7F, G). Furthermore, the results of immunofluorescence staining exhibited that the proportion of M2 macrophages was increased obviously after BMSC-EVs treatment, while the proportion of M1 macrophages was enhanced by pcDNA-PDE1A treatment in the presence of BMSC-EVs (Fig. 7H). All in all, these findings highlighted that BMSC-EVs carrying miR-200b promoted the M2 polarization of macrophages *via* the PDE1A/PKA axis, which further attenuated the formation of PAH.

## Discussion

PAH remains a towering clinical challenge across the globe despite the tremendous advances made in our understanding of the underlying mechanisms and several targeted therapies [27]. The last decade has also witnessed the emergence of BMSC-EVs as important carriers in the modulation of tissue repair and regeneration in lung diseases, including PAH *via* paracrine mechanisms [28]. Accordingly, the current study set out to investigate the specific mechanisms by which BMSC-EVs influence macrophage polarization in PAH, and the obtained findings revealed that BMSC-EVs carrying miR-200b could induce PKA phosphorylation by repressing the expression of PDE1A, whereby promoting M2 polarization of macrophages and ultimately attenuating PAH.

The initial finding in our study demonstrated that miR-200b was poorly-expressed in the lung tissues of rats with PAH, as well as in isolated PASMCs and alveolar macrophages. A large number of studies have uncovered aberrant expression and roles of miRNAs, including miR-200b, in the development of PAH [29, 30]. For instance, miR-200b also exhibits a low expression in the mouse model of cor pulmonale induced by MCT [31]. Likewise, miR-200b was previously shown to attenuate congenital diaphragmatic hernia and rescue pulmonary hypoplasia in rat models [32]. Meanwhile, there is evidence to suggest that miR-200b can further alleviate cellular senescence, as well as inflammatory responses in pulmonary emphysema by targeting ZEB2 [33]. Further expanding on its targeting role, a prior study also indicated miR-200b could ameliorate early pulmonary fibrosis induced by lipopolysaccharide by targeting ZEB1/2 through the p38 MAPK and TGF-β/smad3 axis [34]. All the aforementioned reports underscore that miR-200b confers an important protective role against pulmonary inflammation and other diseases. Partly in accordance with these reports, our findings revealed that miR-200b could be delivered by BMSC-EVs into macrophages to decrease the expression of pro-inflammatory IL-6, TNF-α, IL-1β, and iNOS, while simultaneously promoting the expression of Arg1, which is indicative of M2 macrophage polarization. Macrophages are well-established as a type of immune cell that release inflammatory mediators to regulate immunity. Interestingly, evidence has come to light indicating that BMSCs are capable of repressing the differentiation of macrophages into the M1 phenotypes and attenuating systemic inflammatory response [35]. It is also noteworthy that macrophage M1/M2 imbalance was previously certified as a vital process in the progression of PAH [36, 37]. Furthermore, MSCs have been shown to contribute to reduced expression of iNOS (M1 macrophage marker) and elevated expression of Arg1 (M2 macrophage marker), which was suggestive of M2 macrophage polarization, thus aiding in cardiac repair [38]. Much in line with our findings, another study validated that M2-like macrophage polarization was also promoted by BMSC-EVs, and consequently attenuated ulcerative colitis [39]. Additional in vivo experimentation in our study demonstrated that BMSC-EVs carrying miR-200b exerted a diminishing effect on the RVSP, RVHI and medial thickness of small pulmonary vessels, and thus ultimately alleviated PAH. Excessive proliferation is well-established as the hallmark feature of PAH [40, 41]. Together, these findings and evidence indicate that miR-200b-3p transferred by BMSC-EVs inhibited the proliferation of PASMCs and enhanced their apoptosis, such that BMSC-EVs carrying miR-200b promoted M2 polarization of macrophages to impede the proliferation of PASMCs, thereby contributing to the attenuation of PAH.

Additional experimentation in our study indicated that miR-200b activated the PKA signaling pathway by decreasing the expression of PDE1A. Unsurprisingly, PDE1A has been previously validated as a novel therapeutic target of PAH in a prior study [42]. In addition, up-regulation of the PDE1 family was been documented in pulmonary artery smooth muscle cells in idiopathic PAH lungs, whereas suppression of PDE1 could reverse structural lung vascular remodeling in animal models [43]. Similarly, suppression of PDE1 also exerts a stimulating effect on the response of pulmonary vasodilators to inhaled nitric oxide in animals with acute PAH [44]. Meanwhile, much in accordance with our findings, another study came across increased expression levels of PDE1A in PASMCs obtained from patients with idiopathic PAH [45]. Inherently, PDEs represent a superfamily of enzymes capable of degrading the secondary messengers cAMP and cGMP [46]. Expanding on the same, a prior study reported that PDE1A repression enhanced cGMP signaling and thus promoted the relaxation of rat mesenteric arteries [47]. Besides, PKA serves as the kinase by which cAMP mediates the transcription of various target genes [48]. Intracellular elevations of cAMP and cGMP levels and activation of PKA and PKG have further been associated with the attenuation of PAH [20, 48]. Furthermore, PKA is less phosphorylated in PAH, while also exerting a diminishing effect on RV cardiomyocyte diastolic stiffness in PAH [49]. Recent studies have also indicated that diminished expression of PKA by DP1 inhibition led to the aggravation of vascular remodeling in PAH, involved with mTORC1 signaling [50]. Inhibition of phosphodiesterase III activity can increase cAMP content in cardiomyocytes, activate partial PKA, and phosphorylate phospholamhorin [51]. PDE2A2 regulates the phosphorylation of the MICOS component MIC60 mediated by PKA, thereby regulating the recruitment of Parkin to mitochondria [52]. All the aforementioned reports support our findings regarding the roles of PDE1A and PKA in PAH, but failed to explore the interaction between PDE1A and PKA in PAH. It is also known that CREB, an important transcription factor of the PKA signaling pathway, exerts a significant role in a myriad of human diseases. For instance, lnc-M2-mediated PKA/CREB pathway is capable of regulating M2 macrophage polarization [53]. What’s more, a prior study highlighted that PKA-mediated CREB phosphorylation promotes TNF inhibition and IL-10 induction [54]. In addition, the PKA/CREB pathway in mouse macrophages is closely related to VEGF expression [55], and the latter is closely-associated with the progression of PAH [56, 57]. Therefore, we speculate that PKA may regulate the expression of downstream iNOS and Arg1 through pCREB, thereby influencing the fate and progression of PAH. However, the above mechanism requires further validation in subsequent experiments. Altogether, these findings and evidence indicate that the down-regulation of PDE1A by BMSC-EV-miR-200b induces PKA phosphorylation, which may further exert a suppressive effect on the development of PAH.

## Conclusion

**Fig. 8:**
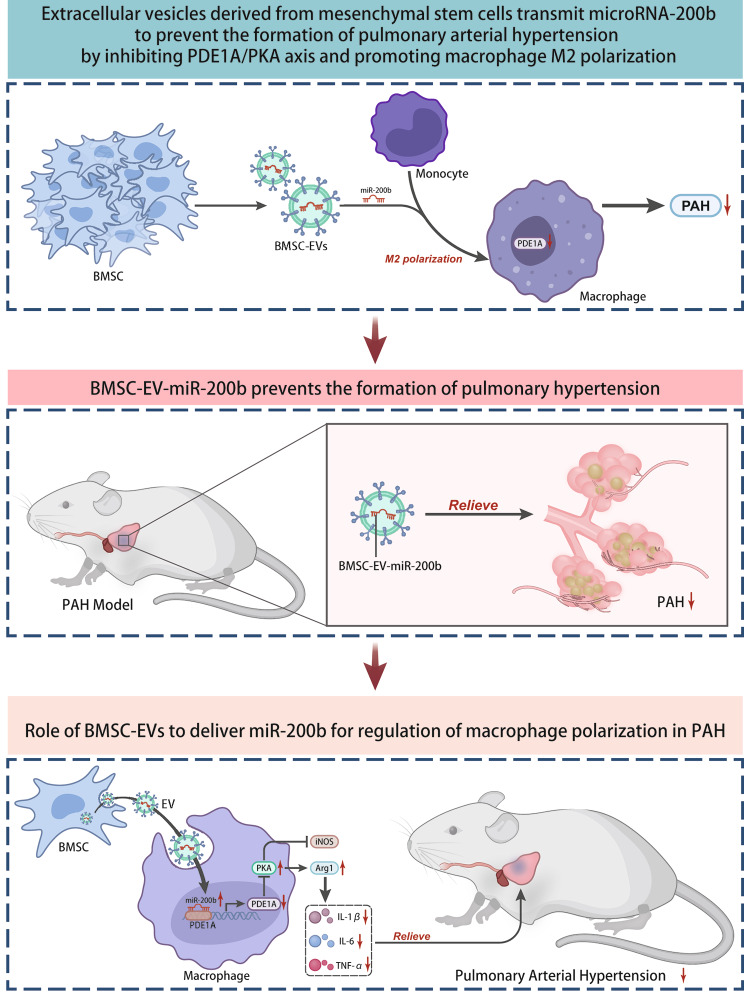
Schematic map of the potential function of BMSC-EVs carrying miR-200b in PAH. BMSC-EVs might deliver miR-200b to alveolar macrophages to inhibit PDE1A and induce PKA phosphorylation, thereby promoting macrophage M2 polarization and ultimately preventing PAH formation

In summary, the current study highlighted that BMSC-EVs might deliver miR-200b to alveolar macrophages to inhibit PDE1A and induce PKA phosphorylation, thereby promoting macrophage M2 polarization and ultimately preventing PAH formation (Fig. 8). However, the accessibility of BMSC-EVs carrying miRNAs remains a hotly-debated research topic and the underlying rules still warrant further investigation. In spite of that the same, our findings indicate that therapeutic strategies and efforts should be directed towards BMSC-EV-miR-200b for the alleviation of PAH.

### Electronic supplementary material

Below is the link to the electronic supplementary material.

Supplementary Material 1

## Data Availability

The data underlying this article will be shared on reasonable request to the corresponding author.

## Abbreviations

PAH: Pulmonary arterial hypertension
MCT: Monocrotaline
EVs: Extracellular vesicles
BMSCs: Bone marrow-MSCs
PKA: Protein kinase
