# Supplementary material for: Mesenchymal stem cell-derived extracellular vesicles prevent the formation of pulmonary arterial hypertension through a microRNA-200b-dependent mechanism
Source: Respir Res. 2023 Sep 27;24:233. doi: 10.1186/s12931-023-02474-7 (PMC10523762; doi:10.1186/s12931-023-02474-7)
Supplement: Supplementary file 1 — Supplementary Material 1 [file 12931_2023_2474_MOESM1_ESM.docx]

**Supplementary Table 1** Information of PAH-related expression microarray

| GEO accession | Coding Type | Platforms | Control Sample | PAH Sample |
| --- | --- | --- | --- | --- |
| GSE21284 | miRNA | GPL10305 | GSE531993 | GSE531995 |
|  |  |  | GSE531994 | GSE531996 |
| GSE113439 | Gene | GPL6244 | GSM3106341 | GSM3106326 |
|  |  |  | GSM3106342 | GSM3106327 |
|  |  |  | GSM3106343 | GSM3106328 |
|  |  |  | GSM3106344 | GSM3106329 |
|  |  |  | GSM3106345 | GSM3106330 |
|  |  |  | GSM3106346 | GSM3106331 |
|  |  |  | GSM3106347 | GSM3106332 |
|  |  |  | GSM3106348 | GSM3106333 |
|  |  |  | GSM3106349 | GSM3106334 |
|  |  |  | GSM3106350 | GSM3106335 |
|  |  |  | GSM3106351 | GSM3106336 |
|  |  |  |  | GSM3106337 |
|  |  |  |  | GSM3106338 |
|  |  |  |  | GSM3106339 |
|  |  |  |  | GSM3106340 |

Note: GEO, gene expression omnibus; PAH, pulmonary arterial hypertension.

**Supplementary Table 2** Primer sequences for RT-qPCR

| Genes | Forward primer | Reverse primer |
| --- | --- | --- |
| miR-200b | 5′-CATCTTACTGGGCAGCATTGGA-3′ | Universal reverse primer |
| IL-6 | 5′-AGAGACTTCCAGCCAGTTGC-3′ | 5′-AGTCTCCTCTCCGGACTTGT-3′ |
| TNF-α | 5′-GGAGGGAGAACAGCAACTCC-3′ | 5′-GCCAGTGTATGAGAGGGACG-3′ |
| IL-1β | 5′-TTGAGTCTGCACAGTTCCCC-3′ | 5′-TCCTGGGGAAGGCATTAGGA-3′ |
| PDE1A | 5′-CATGTGTGGATTGCTCCCGT-3′ | 5′-TTCAGGCGTTGCCACATCTT-3′ |
| PKA | 5′-AGCAGGAGAGCGTGAAAGAG-3′ | 5′-TCCTTGTGCTTCACGAGCAT-3′ |
| U6 | 5′-CTGCACTTATTTCAGAAGCAGATA-3′ | Universal reverse primer |
| iNOS | 5′-GGTCCAACCTGCAGGTCTTC-3′ | 5′-GGTCCATGATGGTCACATTC-3′ |
| Arg1 | 5′-TTTGATGTTGATGGACTGGAC-3′ | 5′-CTGTTCGGTTTGCTGTGATG-3′ |
| GAPDH | 5′-GGCACAGTCAAGGCTGAGAA-3′ | 5′-ATGGTGGTGAAGACGCCAGT-3′ |

Note: RT-qPCR, reverse transcription-quantitative polymerase chain reaction; miR-200b, microRNA-200b; IL-6, interleukin 6; TNF-α, tumour necrosis factor alpha; IL-1β, interleukin-1β; PDE1A, phosphodiesterase 1A; PKA, cAMP dependent protein kinase; iNOS, myo-inositol-1-phosphate synthase; Arg1, arginase 1; GAPDH, glyceraldehyde-3-phosphate dehydrogenase.
